# Effect of Build Orientation on Surface Finish and Hydrodynamic Stability of Inkjet 3D-Printed Microfluidic Channels

**DOI:** 10.3390/polym17131864

**Published:** 2025-07-03

**Authors:** Emanuela Cutuli, Lorena Saitta, Nunzio Tuccitto, Gianluca Cicala, Maide Bucolo

**Affiliations:** 1 Department of Electrical Electronic and Computer Science Engineering, University of Catania, Via Santa Sofia 64, 95125 Catania, Italy; maide.bucolo@unict.it; 2 Department of Civil Engineering and Architecture, University of Catania, Via Santa Sofia 64, 95125 Catania, Italy; gianluca.cicala@unict.it; 3 Department of Chemical Sciences, University of Catania, Viale A. Doria 6, 95125 Catania, Italy; nunzio.tuccitto@unict.it

**Keywords:** additive manufacturing, soft lithography, optical polymer materials, micro-optofluidics, slug flow, optical monitoring

## Abstract

This study examined the effect of build orientation on the surface finish of micro-optofludic (MoF) devices fabricated via a polydimethylsiloxane (PDMS)-based 3D-printing primary–secondary fabrication protocol, where an inkjet 3D-printing technique was implemented. The molds (i.e., primaries) for fabricating the MoF devices were 3D-printed in two orientations: along XY (*Dev-1*) and across YX (*Dev-2*) the printhead direction. Next, the surface finish was characterized using a profilometer to acquire the primary profile of the surface along the microchannel’s edge. The results indicated that the build orientation had a strong influence on the latter, since *Dev-1* displayed a tall and narrow Gaussian distribution for a channel width of 398.43 ± 0.29 µm; *Dev-2* presented a slightly lower value of 393.74 ± 1.67 µm, characterized by a flat and broader distribution, highlighting greater variability due to more disruptive, orthogonally oriented, and striated patterns. These results were also confirmed by hydrodynamically testing the two MoF devices with an air–water slug flow process. A large experimental study was conducted by analyzing the mean period trend in the slug flow with respect to the imposed flow rate and build orientation. *Dev-1* showed greater sensitivity to flow rate changes, attributed to its smoother, more consistent microchannel geometry. The slightly narrower average channel width in *Dev-2* contributed to increased flow velocity at the expense of having worse discrimination capability at different flow rates. This study is relevant for optimizing 3D-printing strategies for the fabrication of high-performance microfluidic devices, where precise flow control is essential for applications in biomedical engineering, chemical processing, and lab-on-a-chip systems. These findings highlight the effect of microchannel morphology in tuning a system’s sensitivity to flow rate modulation.

## 1. Introduction

Additive manufacturing (AM) is a fabrication process for producing 3D objects from 3D model data, i.e., CAD (computer-aided design) [1], by adding material layer-by-layer until the object is completed [2,3,4]. In accordance with the ASTM F2792-12a standard [5], AM processes are classified into seven categories according to their operating principle [6] and present different properties in terms of resolution, accuracy, manufacturing rate, porosity, implemented materials, mechanical features, and so on. AM techniques have been used to create final products with custom shapes quickly, accurately, and consistently, even those with a high degree of geometry complexity [7,8,9,10]. Among the existing AM techniques, in the material jetting (MJ) process, tiny droplets of liquid material are sprayed layer-by-layer onto a surface and then hardened using UV light. This process allows for producing highly detailed and accurate parts, making it particularly suitable for applications that require precision and fine features [11,12,13]. In microfluidic device fabrication, material jetting offers several key advantages. Its high resolution and smooth surface finish are ideal for creating tiny channels that guide fluid flow. MJ 3D printing is also valuable in the production of molds for primary–secondary applications [14,15]. It allows for the rapid creation of detailed and complex mold geometries without the need for traditional machining. This speeds up the prototyping process, reduces manufacturing costs [16], and has a precision that produces high-quality molds that can be used to replicate parts with great accuracy. However, it must be taken into account that the quality of the manufactured product, such as the surface finish consistency, is affected by several process factors: building part orientation, part location on the building platform, printhead positioning gantry velocity, printhead movements, nozzle tip, and so on [17,18,19,20]. However, only few studies have been carried out to understand how these parameters affect the surface finish of functional parts manufactured via MJ 3D printing. While these studies have investigated how the layer thickness [21,22], the part build orientation [21,23,24,25,26,27,28], the finish type or build style (matte or glossy) [21,22,23,25,27], the size of the part [25], and the implemented post-processing protocol [25] influence the part’s surface roughness or mechanical properties, to the best of our knowledge, no mention has been made regarding the final functionality in terms of the hydrodynamic behavior properties of slug flow in micro-optofluidic (MoF) devices fabricated via the MJ 3D printing of polydimethylsiloxane (PDMS)-based primary–secondary approach.

Microfluidic systems have emerged as powerful platforms for precise fluid manipulation at the microscale, enabling advancements in chemical synthesis [14,29], biological analysis [30], and medical diagnostics [31,32]. Among the various flow regimes observed in microchannels, slug flow is particularly advantageous due to its wide employment in real-world multiphase flow applications, including heat transfer and micromixing systems [33], droplet-based microfluidics [34], lab-on-a-chip (LoC) systems [35], and microreactors [36]. The slug flow regime in microfluidic channels is characterized by alternating segments (*slugs*) of immiscible liquids or gas [37,38,39,40]. However, morphological irregularities within the microchannels can disrupt the consistent formation and spacing of these slugs [41,42]. When inkjet 3D printing is used to fabricate microfluidic structures, it can introduce layer lines and surface textures that vary depending on the print path and build orientation [43,44]. These striated surface patterns directly influence the resulting surface finish, playing a significant role in influencing the slug flow behavior within microfluidic channels. In particular, when the layer lines are aligned parallel to the flow direction, they tend to create smoother flow paths with less disruption to slug integrity and motion. In contrast, when the layer lines are orthogonal to the flow direction, they act as periodic surface ridges, resulting in jagged channel walls, bends, sudden expansions or contractions, and non-uniform cross-sections [45]. These can induce flow resistance and promote disturbances that can lead to premature slug breakup or coalescence. These directional surface textures alter local hydrodynamics and interfacial interactions, ultimately affecting slug length, velocity, and formation frequency [46,47].

In this context, the novelty of this study is investigating for the first time how build orientation impacts the surface finish and the hydrodynamic slug flow stability in micro-optofluidic (MoF) devices manufactured using a PDMS-based primary–secondary fabrication process, in which the primaries are produced via inkjet 3D printing. Two build orientations were considered: one aligned with the XY plane (*Dev-1*) and another across the YX plane (*Dev-2*) relative to the printhead direction. The surface finish was characterized by using profilometry to obtain the primary surface profile along the microchannel edge. Furthermore, the hydrodynamic behavior of the two devices was evaluated by means of optical signal acquisition under an air–water slug flow regime through an extensive experimental campaign, analyzing how slug flow periodicity varied with flow rate and build orientation. A key advantage of the proposed approach lies in its reliance on optical signal acquisition, which enables the high-frequency monitoring of slug flow dynamics. This method allows for the precise and non-invasive mapping of evolving multiphase structures and their interactions within the microchannel, providing valuable insights into transient phenomena. Together, these findings underscore the importance of microchannel morphology and having a monitoring methodology for tuning a system’s sensitivity to flow rate modulation.

This paper is organized as follows: Section 2 discusses the materials and methodologies, including the materials employed (Section 2.1), the MoF device design, working principles, the manufacturing protocol (Section 2.2), the methodologies used for the surface finish quality assessment (Section 2.3 and Section 2.4) and its effect on hydrodynamic slug flow stability (Section 2.5). The results are discussed in Section 3, while the conclusions and future developments are presented in Section 4.

## 2. Materials and Methods

### 2.1. Materials

The mold for the polydimethylsiloxane (PDMS) casting was fabricated using a photopolymer VeroWhitePlus RGD835 (OVERMACH S.p.A., Parma, Italy), a proprietary blend of acrylate monomers and photoactivators. The PDMS used for device fabrication was SYLGARD™184 Silicone Elastomer kit (Dow Corning, Midland, MI, USA), consisting of a base and curing agent, purchased from Farnell Italia S.R.L. (Milan, Italy).

### 2.2. MoF Device: Design, Working Principle, and Manufacturing Protocol

**Figure 1:**
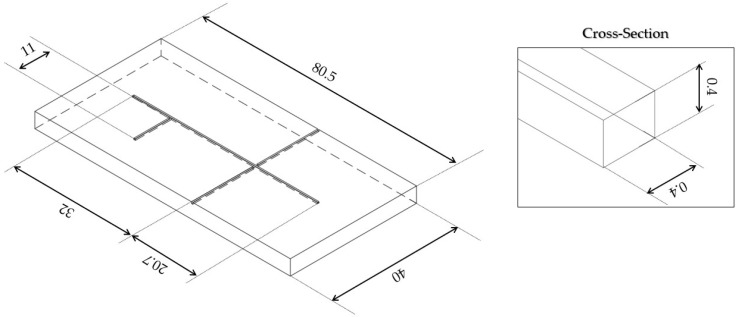
Axonometric view of the 3D-printed mold (on the **left**). The inset (on the **right**) shows a magnification of the microchannel and the fiber’s slot. Sizes are expressed in [mm].

The two micro-optofluidic (MoF) devices investigated in this study featured a T-junction formed by two intersecting microchannels, enabling the generation of the slug flow. The setup included two inlets for introducing fluids into the microchannel and a single outlet for their discharge. An actuation optical fiber was inserted into the device to direct a light beam toward the region of interest within the microchannel. As the light interacted with the alternating fluids in the slug flow, the resulting optical signals were collected by a detection optical fiber positioned on the opposite side of the channel (see Figure 1).

The MoF device was designed to detect slug flow composed of immiscible fluids, operating based on the principles of light absorption. The interaction between the incident laser beam and each fluid depends on the fluid’s refractive index, which influences the amount of light transmitted. As a result, the detected optical signal varies in amplitude, depending on the interacting fluid at a given moment. Specifically, the signal exhibits a square-wave pattern, with two distinct levels corresponding to the two different fluids in the slug flow.

**Figure 2:**
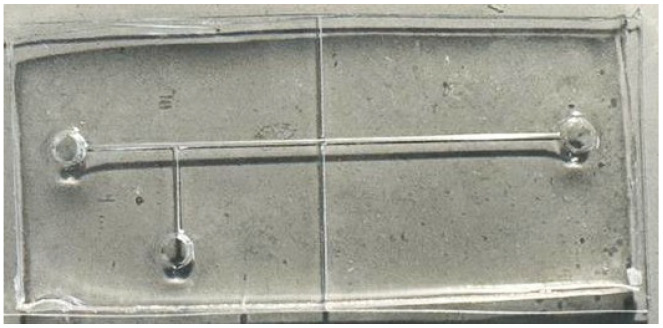
Final assembled PDMS MoF device.

The MoF devices were fabricated using PDMS through a primary–secondary approach based on inkjet 3D printing, described in detail elsewhere [14,40]. Briefly, the fabrication process followed a multi-step workflow: (i.) mold design in Autodesk®Fusion 360 (v.2.0.17721) software; (ii.) slicing and G-code generation with Objet ${\mathrm{Studio}}^{TM}$ software (v.9.2.11.6825); (iii.) mold fabrication using a PolyJet 3D printer. The 3D printer was a Stratasys Objet260 Connex1 (Stratasys, Los Angeles, CA, USA). PDMS was prepared by mixing the silicone elastomer base and curing agent in a 10:1 ratio for the device layer and a 5:1 ratio for the cover layer, which was then degassed, poured into the mold, and cured at room temperature for 48 h. The cured parts were demolded and bonded using a reversible process, with both surfaces plasma-treated for 3 min using a Plasma Bonding Pen (Elveflow, Paris, France) to enhance adhesion. The final bonded device is shown in Figure 2.

### 2.3. Quality Control of Consistency of MoF Device’s Surface Finish

Although the settings used for the printing profile for the mold manufacturing ensured the optimum surface roughness results (as confirmed in previous studies [26]), in terms of smooth surface finish and fine detail resolution, the final outcome was not entirely free from surface artifacts. In fact, due to the layer-by-layer deposition process and the nature of using multiple inkjet printheads, a wave-like pattern or striated surface patterns are often observed on the surfaces of printed objects [48]. When a microchannel is 3D-printed, this surface waviness due to inkjet head layering (known as an artifact in this 3D-printing technique), when properly combined with its build orientation, may significantly affect its final dimensional accuracy. Thus, when a microchannel is oriented parallel to the direction of printhead travel (i.e., along the *X*-axis), the printhead deposits material along the length of the channel. This orientation tends to provide superior dimensional accuracy and surface consistency, as the channel structure is printed in continuous, uninterrupted passes. Since each pass of the printhead aligns with the channel axis, the chances of errors caused by inter-pass misalignment or swath boundaries are reduced. As a result, the sidewalls along the channel length are smoother, and the channel maintains a more uniform cross-section. Conversely, orienting a microchannel orthogonally (i.e., along the *Y*-axis) to the printhead movement introduces several dimensional challenges. In this case, the printhead must create the channel by interleaving multiple swaths, each slightly offset. The resulting surface can exhibit visible striations or ridges, potentially causing asymmetry in the channel cross-section.

**Figure 3:**
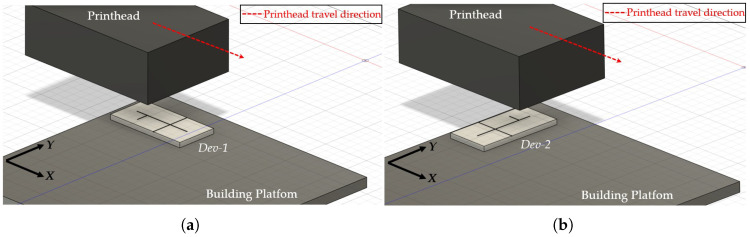
Schematic of the microchannel (mold) orientation with respect to the printhead travel direction: (**a**) parallel and (**b**) orthogonal to the direction of printhead travel.

**Figure 4:**
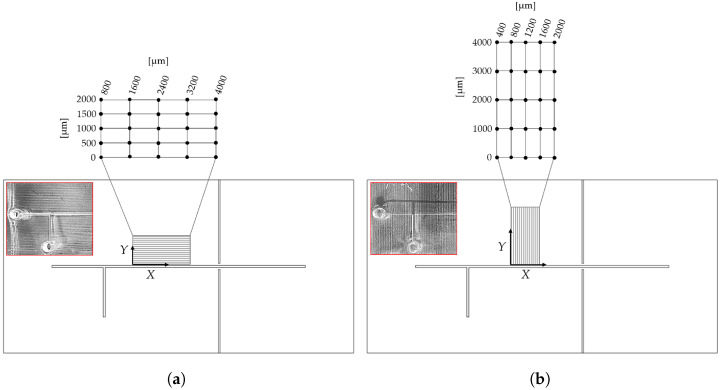
X− and Y−*Coordinates* selected for the observations collection in **Step 3** for (**a**) *Dev-1* and (**b**) *Dev-2*. In the red inset, a picture of the final PDMS device highlights the effect of the printhead’s travel direction.

To examine how the mold build orientation affected the surface consistency of the MoF device’s microchannels, the following procedure was implemented:

- **Step 1**. *Definition of mold manufacturing.* Two different molds were 3D-printed: the 1^*st*^ one with the microchannel oriented along (XY) the printing head direction (see Figure 3a), named *Dev-1*; the 2^*nd*^ one with the microchannel oriented across (YX) the printing head direction (see Figure 3b), named *Dev-2*.
- **Step 2**. *Observation collection.* For each 3D-printed mol, the primary profile was acquired by using a KLA Tencor®P-7 Stylus Profiler (Gambetti, Milan, Italy) at a scan speed of 200 µm/s, an *X* resolution of 4 µm, and a *Y* resolution of 50.5 µm. For each mold a surface of 2000 × 4000 µ$m^{2}$, oriented as represented in Figure 4a,b for *Dev-1* and *Dev-2*, respectively, was mapped.
- **Step 3**. *ANOVA study.* For each 3D-printed mold, the surface consistency was examined as follows: a replicated general factorial design was studied by means of a third-order model (*p*-value < 0.0001), which was the most significant from the sequential model’s sum of squares (Type I) test, with the aim of finding a suitable approximation for the true functional relationship between the primary profile and the set of independent variables. In detail, the investigated factors (independent variables) for the experimental design are reported below:
  - X−Coordinates (*factor A*)—numerical factor varied among five different levels (a=5) corresponding to {800;1600;2400;3200;4000} µm for *Dev-1* and {400;800;1200;1600;2000} µm for *Dev-2*.
  - Y−Coordinates (*factor B*)—numerical factor varied among five different levels (b=5) corresponding to {0;500;1000;1500;2000} µm for *Dev-1* and {0;1000;2000;3000;4000} µm for *Dev-2*.
  The number of replications was fixed at n=5, for a total number of N=a×b×n=125 experimental runs. The investigated response (dependent variable) for the experimental plan was the height (expressed in [µm]) related to the acquired primary profile. Once the observations for the latter response were collected, a further analysis of variance (ANOVA) was performed to examine the statistical significance of each investigated factor together with their potential interaction. The experimental plans are reported in Table 1 and Table 2 for *Dev-1* and *Dev-2*, respectively. It was necessary to consider two different experimental plans for the two devices, since the used profilometer was characterized by two diverse resolutions along *X* and *Y*.

### 2.4. Morphological Analysis: Optical Microscopy

**Figure 5:**
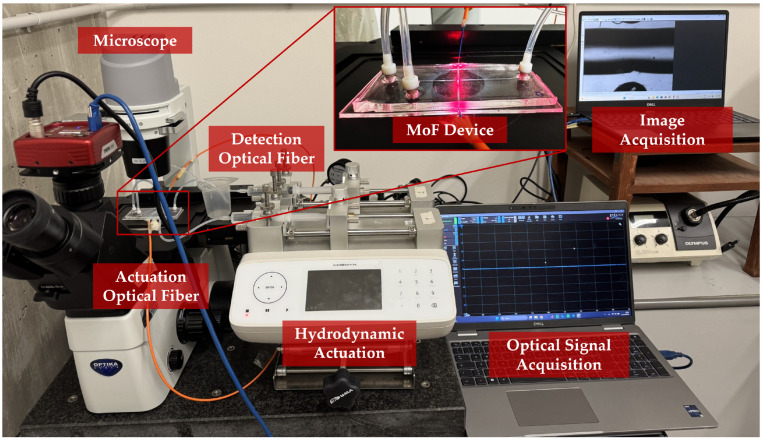
Real picture of the experimental setup, including the fluidic connections, optical alignment, and support structure used during the experimental plan.

The optical micrographs of both the *Dev-1* and *Dev-2* microchannels were acquired using an Optika IM-300 optical Microscope (Optika, Ponteranica, Italy) in the bright-field observation mode, which was equipped with a WD pre-centered condenser (N.A. 0.30, W.D. 72 mm) and a 4× magnification objective lens (PLN, Olympus, Tokyo, Japan). Furthemore, a CCD camera (340M Fast Frame, Thorlabs, Newton, NJ, USA) with a resolution of 640 pixels × 480 pixels (pixel size of 7.4 µm, square) was used to acquire the optical images by connecting the camera to the PC by means of a USB. The optical microscope setup is shown in Figure 5.

### 2.5. Impact of Build Orientation on Slug Flow’s Hydrodynamic Stability: Experimental Setup and Campaign

A real picture of the system employed to assess the impact of build orientation on the slug flow’s hydrodynamic stability is provided in Figure 5. It consisted of the components as follows: (i.) a hydrodynamic system for introducing fluid samples into the micro-optofluidic device; (ii.) an optical actuation system with a laser source coupled via an actuation optical fiber; (iii.) the MoF device; (iv.) an optical detection system using a photodiode connected to an output optical fiber; (v.) a computer equipped with dedicated software for acquiring the optical signals.

To generate the slug flow within the MoF device, deionized water and air were simultaneously injected through the two inlets of its T-junction geometry. This was achieved using two Fusion 4000 Independent Channels Syringe Pumps (Chemyx, Stafford, TX, USA), one loaded with deionized water and the other with air. These pumps enabled the precise control of the flow rate values and were connected directly to the device inlets. For the optical actuation, a NovaPro 660-125 laser system (RGB Lasersystems, Kelheim, Germany) was used, operating at a wavelength of 660 nm and a power of 1 mW. The laser light was delivered to the device via an actuation optical fiber with a 365 µm core diameter, inserted into the device’s actuation slot. Optical detection was performed using a 365 µm diameter output optical fiber connected to a PDA100A photodiode (Thorlabs, Newton, NJ, USA) with a gain setting of 40 dB, enabling the detection of light intensity variations. The photodiode output was recorded using a PicoScope 2204A USB oscilloscope (Pico Technology, Cambridgeshire, UK), operated at a sampling rate of 1.5 kHz for high-resolution signal acquisition.

The post-processing of the acquired optical signals was carried out in MATLAB (v.R2023b, MathWorks®), as described in detail elsewhere [37,38].

The experiments were conducted under ambient laboratory conditions. The laboratory temperature was maintained between 20 °C and 25 °C and monitored during the testing. Under these conditions, the percentage changes in water and air viscosity were minimal, i.e., about 11% and 2.8%, respectively, thus were not expected to significantly alter the slug flow behavior under the tested conditions, and any variation could be considered negligible for the purposes of this study. Viscosity parameters were assumed to be equal to 1.0016 mPa · s at 20 °C for water and 0.0181 mPa · s at 20 °C for air.

The investigated response for the experimental plan was the mean period (*T*) associated with complete slug flow passage. It was calculated starting from the detected square-wavelike optical signal $S_{opt}\left(t\right)$ in the frequency domain. Thus, the frequency of the highest peak was extracted from the spectrum $S_{opt}\left(f\right)$ to evaluate the fundamental component (f), and the mean period (T) was calculated as the reciprocal of the latter parameter.

To investigate how the mold build orientation affected the surface consistency of the MoF device’s microchannels and, consequently, their hydrodynamic stability in the slug flow formation period, a replicated general factorial design was used. The investigated factors (independent variables) for the experimental design are reported below:

- *Device (factor A)*—categorical factor varied among two levels (a=2), which were {*Dev-1*, *Dev-2*};
- *Flow rate (factor B)*—quantitative factor varied among five levels (b=5), which were {0.025,0.05,0.1,0.2,0.3} mL/min.

The number of replications was set at n=3, for a total of N=a×b×n=30 experimental tests. Moreover, it was taken into account that the experimental tests were not completely randomized. Thus, the experiments were divided into 3 different blocks, each block corresponding to a certain replication value (i.e., n=1,2,3). The latter approach of considering blocks aimed to balance out the effects of the replications, hence eliminating its influence on the analysis. The experimental plan is reported in Table 3.

## 3. Results and Discussion

**Figure 6:**
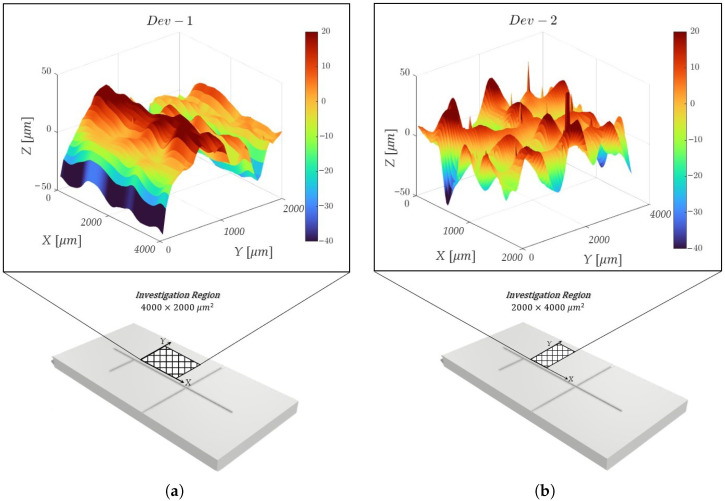
Surface profile acquired with a profilometer in the investigated region: (**a**) *Dev-1* and (**b**) *Dev-2*.

The 3D surface reconstructions of the primary profile [µm] mapped through profilometry for *Dev-1* and *Dev-2* are reported in Figure 6. *Dev-1* presented an average surface roughness ($R_{a}^{\left(1\right)}$) equal to [7.28; 7.41] µm (expressed as the 95% confidence interval), while it was equal to [6.60; 6.70] µm for *Dev-2*. In detail, $R_{a}^{\left(1\right)}$ represents the arithmetic average of the absolute values of the roughness profile values, and it was evaluated as follows:

$$R_{a}^{\left(1\right)}=\frac{1}{n}\sum_{i=1}^{n}\left|y_{i}\right|\;\;\left(Method\;1\right)$$

where $|y_{i}|$ is the absolute value of each collected primary roughness profile value, and *n* is the total number of collected observations. By considering the absolute value of the arithmetic mean of all profile values (named as $R_{a}^{\left(2\right)}$), it was evaluated as follows:

$$R_{a}^{\left(2\right)}=\frac{1}{n}\sum_{i=1}^{n}y_{i}\;\;\left(Method\;2\right)$$

which ranges from 0.65 up to 2.09 µm. In the end, the individually collected primary roughness profile observations ranged within the interval [0.1–19.9] µm (*Method 3*), having excluded the outliers from the collected dataset (visible in Figure 6). The obtained results are consistent with the findings previously reported for state-of-the-art methods (see Table 4) for 3D-printed parts (microchannels and benchmarks) by using different materials, 3D-printing techniques, and printing settings.

Figure 6 shows that *Dev-1* presents a uniform microchannel edge profile (along the *X*-axis), while striated surface patterns are pronounced along the *Y*-axis (see Figure 6a). This was due to the mold orientation during the 3D-printing process, i.e., with the microchannel oriented along XY, the printing head direction (as described in Section 2.3). Conversely, even the microchannel edge profile (along the *X*-axis) for *Dev-2* shows striated surface patterns (see Figure 6b). Once again, the latter jagged morphology along the microchannel profile was due to the mold orientation during the 3D printing process, i.e., with the microchannel oriented across the printing head direction (YX) (as described in Section 2.3). The findings discussed so far were also confirmed by the implemented procedure by examining the effects of mold build orientation on the surface consistency of the MoF device’s microchannel (presented in Section 2.3).

**Figure 7:**
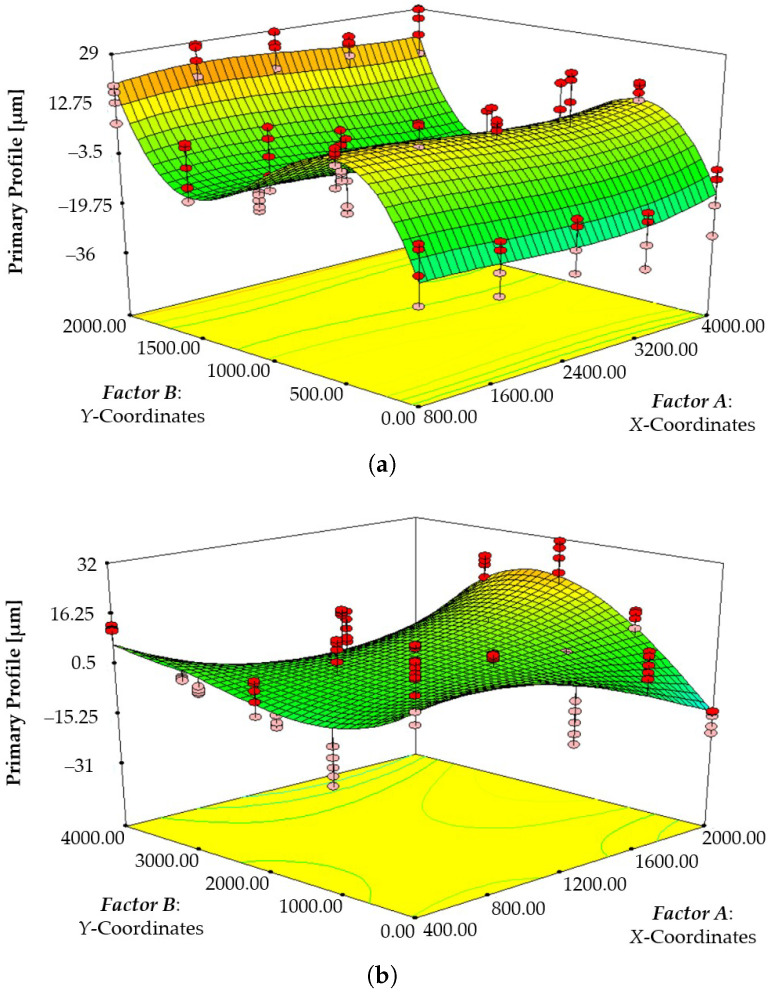
A three-dimensional response surface showing the expected primary profile [µm] as a function of the X− and Y−Coordinates for the surfaces of (**a**) *Dev-1* and (**b**) *Dev-2*.

In detail, the ANOVA tables for the primary response profile are reported in Table 5 and Table 6 for *Dev-1* and *Dev-2*, respectively. The three-dimensional response surfaces showing the expected primary profile [µm] as a function of the X− and Y−Coordinates for the surface of *Dev-1* and *Dev-2* are reported in Figure 7a and Figure 7b, respectively.

From the obtained results it was found that the Y−Coordinates (*factor B*) was a influential factor (*p*-value <0.0001) for *Dev-1*, which was a strong predictor of its primary surface profile. Furthermore, even $B^{3}$ (the cubic term for *factor B*) was highly significant (*p*-value <0.0001), which suggested that the cubic relationship with the Y−Coordinates played a crucial role in explaining the response variable. Conversely, the X−Coordinates (*factor A*) was the influential factor (*p*-value = 0.0454 < 0.05) for *Dev-2*, thus being a strong predictor of its primary surface profile. Moreover, it must be noted that while the first order of *factor B* is not significant (*p*-value > 0.0001), the quadratic $B^{2}$ and cubic $B^{3}$ terms are highly significant (*p*-value <0.0001), which are due to the S-shaped surface response (with inflection points), as also observed for the response surface curves with respect to *factor B*, as shown in Figure 7b. This also justifies the significance of the interaction of the $A^{2}B$ and $AB^{2}$ terms (*p*-value <0.0001). It must be also highlighted that approximately $R^{2}=76.23\%$ and $R^{2}=55.85\%$ of the variability in the primary profile [µm] response (dependent variable) is explained by the model for *Dev-1* and *Dev-2*, respectively, i.e., by the influential factors. Although the latter $R^{2}$ value for *Dev-2* surface characterization represents a moderate proportion of explained variance, the model is statistically significant overall (*p*-value < 0.001) and successfully identifies the key factors and interactions that influence the surface profile. Furthermore, the primary objective of this modeling effort is not to achieve high predictive accuracy but rather to enhance the understanding of the physical behavior of the system. In the end, no anomalies were identified from checkingf the adequacy of models related to *Dev-1* and *Dev-2* using ANOVA.

**Figure 8:**
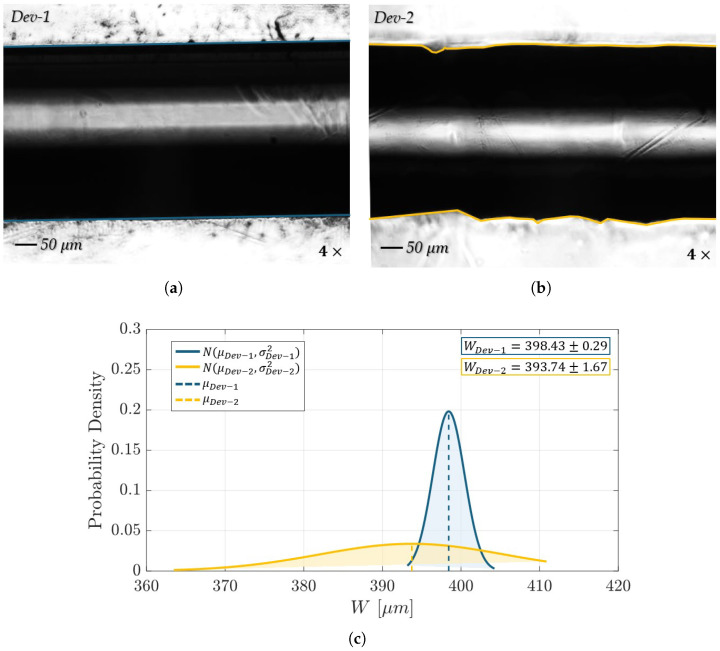
Grayscale frame of the microfluidic channels overlaid with the extracted microchannel profile (magnification: 4×): (**a**) *Dev-1* and (**b**) *Dev-2*. (**c**) Superimposition of the distribution of 50 measurements of the microchannel widths (W [μm]), with a normal distribution curve interpolated for the data for *Dev-1* (blue curve) and *Dev-2* (yellow curve).

Figure 8 illustrates a morphological comparison between the microfluidic devices (*Dev-1* and *Dev-2*), highlighting the channel width and microchannels boundaries profiles. For *Dev-1* (see Figure 8a), the tracked microchannel profile appears smoother and more uniform, whereas *Dev-2* (see Figure 8b) exhibits noticeable irregularities along the microchannel walls. This difference is attributed to the orientation of the striated surface patterns with respect to the microchannel: In *Dev-1*, the striated patterns are predominantly aligned parallel to the microchannel direction, which preserves profile continuity. In contrast, *Dev-2* shows striated patterns oriented more orthogonally to the channel, leading to a more jagged and less-uniform appearance. Further evidence of this can be seen in Figure 8c, showing a superimposition of probability density functions of 50 measurements of the microchannel width (*W* [µm]), with a normal distribution curve interpolated for the data of *Dev-1* (blue curve) and *Dev-2* (yellow curve). *Dev-1* displays a tall and narrow Gaussian distribution with a mean width of 398.43 µm and a standard error of 0.29 µm, reflecting high dimensional uniformity. *Dev-2*, on the other hand, is characterized by a slightly lower mean width of 393.74 µm as well as a flat and broader distribution, with the standard error being equal to 1.67 µm, highlighting greater variability, likely induced by more disruptive, orthogonally oriented striated patterns.

**Figure 9:**
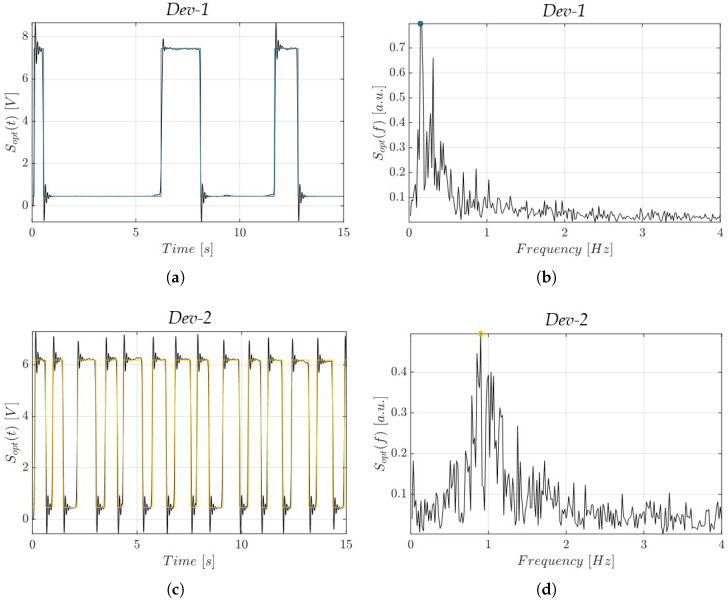
Acquired optical signals in the time domain ($S_{opt}\left(t\right)$) and in the frequency domain ($S_{opt}\left(f\right)$) at *P* = 1 mW and FR = 0.05 mL/min: (**a**,**b**) *Dev-1* (light blue) and (**c**,**d**) *Dev-2* (yellow).

**Figure 10:**
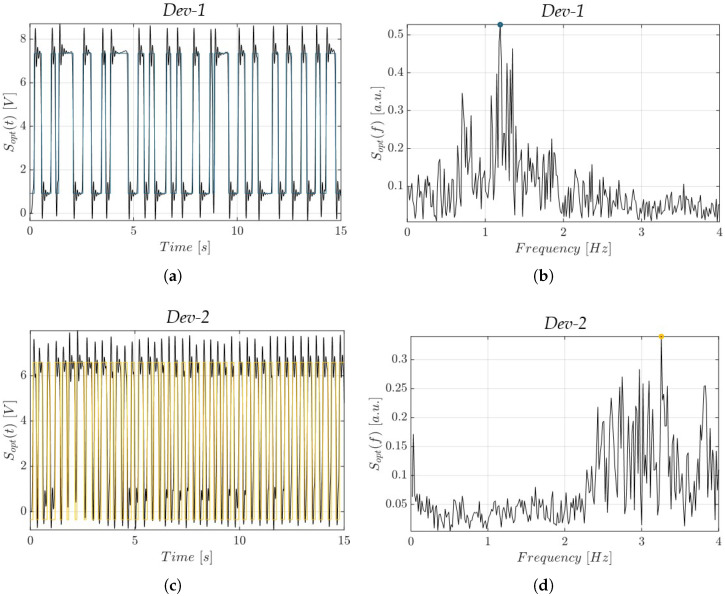
Acquired optical signals in the time domain ($S_{opt}\left(t\right)$) and in the frequency domain ($S_{opt}\left(f\right)$) at *P* = 1 mW and FR = 0.2 mL/min: (**a**,**b**) *Dev-1* (light blue) and (**c**,**d**) *Dev-2* (yellow).

The observed morphological differences between the devices had a direct impact on the hydrodynamic behavior of the slug flow within the microchannels, as investigated through optical signal acquisition. The optical signals in the time domain $S_{opt}\left(t\right)$, along with their corresponding frequency spectra $S_{opt}\left(f\right)$, are presented in Figure 9 and Figure 10, for *Dev-1* and *Dev-2*, under an operative condition of *P* = 1 mW, with a flow rate FR = 0.05 mL/min and FR = 0.2 mL/min, respectively. For the sake of brevity, only these representative conditions are shown; the complete data from all tested scenarios are available upon request from the authors. Under both conditions, *Dev-2* exhibited a greater number of complete air–water oscillations within the 15 s observation window compared to *Dev-1* (see Figure 9a vs. Figure 9c and Figure 10a vs. Figure 10c). This was further supported by the fundamental frequency identified from the spectral peaks in $S_{opt}\left(f\right)$, which consistently indicated that *Dev-2* operated at a higher slug flow frequency than *Dev-1* under identical conditions.

These differences in slug flow behavior directly correlate with the previously identified morphological characteristics of the two devices. Indeed, *Dev-1* exhibits smoother and more uniform channel profiles, with a narrow width distribution and striated patterns oriented parallel to the flow direction. In contrast, *Dev-2* shows greater surface irregularity and a broader width distribution, along with a slightly smaller mean channel width. This reduced cross-sectional area increases flow velocity under constant volumetric flow rate conditions, promoting more frequent slug formation and breakup.

The ANOVA table for the *T* response is reported in Table 7. In this case, both *factor A* and *factor B*, together with their *interaction, AB*, are factors influencing (*p*-value <0.0001) the investigated response. This result suggest that, in accordance with the morphological findings, in terms of the striated surface patterns with respect to the microchannel direction, the two fabricated molds with different orientations with respect to the printing head direction (along and across) show different hydrodynamic behavior. The latter is also affected, as expected, from the set flow rate value for the two fluids making up the slug flow. Similar results were also found in previous work, where tailored ZnO nanostructured coatings, as microchannel surface functionalization, allowed the modification of the microchannel morphology, fine tuning the fluid dynamics inside a microchannel [15].

In the end, no anomalies were found when checking model adequacy, and from $R^{2}=0.9932$ it can be inferred that almost the entire response variation was due to the independent factors variation.

**Figure 11:**
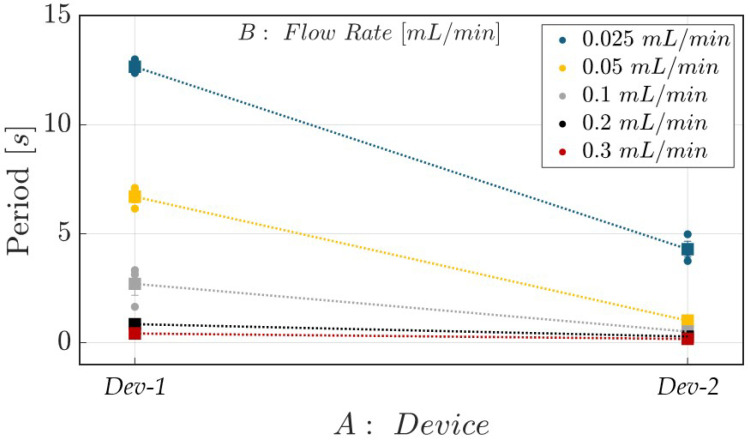
Effects diagram of the investigated mean period (*T*) associated with complete air–water slug flow passage for both *Dev-1* and *Dev-2* at various FR values.

The same conclusions can be drawn by considering the effects diagram for the investigated mean period *T* reported in Figure 11. The plot confirms that *Dev-2* consistently exhibits shorter slug periods compared to *Dev-1* across all tested flow rate values. An additional observation is that *Dev-1* demonstrates greater sensitivity to variations in flow rate, as evidenced by the wider spread in the slug period values across the tested conditions. In contrast, *Dev-2* shows a more compressed response span, with slug periods changing less distinctly between flow rate values. This improved discriminatory capability of *Dev-1* is the result of its smoother and more-uniform microchannel geometry, which enables more consistent flow patterns and minimizes the stochastic effects introduced by surface irregularities. Consequently, changes in the flow rate more directly influence the hydrodynamic behavior of *Dev-1*, making it more responsive to minor variations in operational parameters. In contrast, the more irregular profile of *Dev-2* introduces additional sources of disturbance and local turbulence. These effects hide the impact of incremental changes in the flow rate. Additionally, the slightly narrower average channel width in *Dev-2* contributes to an increased flow velocity, promoting frequent slug formation. These findings collectively highlight the effect of microchannel morphology not only in determining slug flow frequency but also in tuning the system’s sensitivity to flow rate modulation. However, the latter strategy, by properly modulating the micrometric deviations of the surface morphology, can be employed at lower flow rate values (i.e., FR ≤ 0.2 mL/min), since *Dev-1* and *Dev-2* ensure similar hydrodynamic behavior when this threshold value is exceeded (see the black and red lines in Figure 11). This outcome is supported by earlier studies, which showed that more consistent hydrodynamic behavior, specifically regarding the frequency peak, was observed in two-phase flow systems when higher flow rate values were applied [52]. Additionally, it was demonstrated that such hydrodynamic processes exhibited greater periodicity under increased flow rate conditions, reaching up to 1 mL/min [53].

These findings support the general conclusion that smoother and more-uniform microchannel geometries enhance the sensitivity and stability of air–water slug flow, although this effect is strongly influenced by fluid properties, particularly viscosity and density. High-viscosity fluids stabilize slug formation and amplify the benefits of geometric consistency, while low-viscosity fluids lead to faster, less-stable slugs that remain sensitive to flow disturbances. Similarly, fluid density plays a key role: higher-density liquid phases increase the inertial resistance in irregular channels (i.e., *Dev-2*), resulting in more unpredictable slug elongation and flow variability. In contrast, low-density gas phases are more prone to velocity fluctuations and irregular slug breakup. As a result, flow stability and measurement precision deteriorate more significantly in geometrically inconsistent devices. These trends are consistent with prior studies [54,55,56], which emphasize the role of viscous and inertial forces, captured through dimensionless numbers such as Ca, Oh, and We, in governing slug flow regimes. Overall, our results confirm that smoother microchannels (i.e., *Dev-1*) maintain more-regular and controllable flow behavior, particularly under varying flow rates.

## 4. Conclusions

In this work the effect of build orientation on the surface finish and hydrodynamic stability in microfluidic channels fabricated via inkjet 3D printing was investigated. For this purpose two molds (i.e., primaries) for MoF devices were 3D-printed in two orientations: (*Dev-1*) along XY and (*Dev-2*) across YX of the printhead direction. The primary surface profile along the microchannel’s edge was characterized using a profilometer. Additionally, to assess the effect of the striated surface patterns on hydrodynamic stability, the MoF devices were tested in terms of air–water slug flow by estimating the oscillation mean period (*T*) trend with respect to the imposed flow rate value and build orientation. The hydrodynamic behavior was monitored by means of optical signal acquisition through an extensive experimental campaign, enabling the high-frequency mapping of the slug flow periodicity across different flow rate values and build orientations.

The findings demonstrate that the build orientation significantly impacts the microchannel morphology. Specifically, *Dev-1* exhibits a tall, narrow Gaussian profile for its channel width, measured at 398.43 ± 0.29 µm, indicating a more uniform structure. In contrast, *Dev-2* shows a slightly reduced channel width of 393.74 ± 1.67 µm, with a broader and flatter distribution, suggesting higher variability caused by more disruptive, striated patterns oriented orthogonally. As a result, *Dev-1* responds more sensitively to variations in the flow rate value due to its smoother and more consistent microchannel geometry. Conversely, the marginally narrower average width value in *Dev-2* leads to an increased flow velocity but compromises its ability to distinguish between different flow rates. These results underscore how the microchannel structure plays a critical role in modulating the system’s responsiveness to flow rate changes and, thus, pave the way for examining the development of innovative solutions within the field of *f*AM (functional AM) to modify the surface finish of microchannels by ad hoc tailoring the texture on micro- and nanoscales. Additionally, although this study focused on straight channels and low-viscosity fluids, the observed effects of build orientation on surface morphology and flow behavior are likely relevant to more complex geometries (e.g., spiral channels) and high-viscosity or non-Newtonian fluids. Future work will explore these scenarios to assess how striated surface patterns influence flow dynamics under more demanding conditions.

## Figures and Tables

**Table 1:** Experimental plan for primary profile acquired for *Dev-1*: factors and levels.

Factor	Symbol	Type	Unit	Levels	Level I	Level II	Level III	Level IV	Level V
X− *Coordinates*	A	Numerical	[µm]	a=5	800	1600	2400	3200	4000
Y− *Coordinates*	B	Numerical	[µm]	b=5	0	500	1000	1500	2000

**Table 2:** Experimental plan for primary profile acquired for *Dev-2*: factors and levels.

Factor	Symbol	Type	Unit	Levels	Level I	Level II	Level III	Level IV	Level V
X− *Coordinates*	A	Numerical	[µm]	a=5	400	800	1200	1600	2000
Y− *Coordinates*	B	Numerical	[µm]	b=5	0	1000	2000	3000	4000

**Table 3:** Experimental plan for slug flow formation period: factors and levels.

Factor	Symbol	Type	Unit	Levels	Level I	Level II	Level III	Level IV	Level V
*Device*	A	Categorical	[mL/min]	a=2	*Dev-1*	*Dev-2*	[-]	[-]	[-]
*Flow Rate*	B	Categorical	[-]	b=5	0.025	0.050	0.1	0.2	0.3

**Table 4:** Comparison of roughness measurements of parts 3D-printed by means of different 3D-printing techniques and materials. BJP: binder jetting process. FDM: fused deposition modeling. SLS: selective laser sintering. PJ: PolyJet. SL: stereolithography.

Printing Method	3D Printer	Material	Roughness [µm]	Measurement Method	Reference
Inkjet	Projet3510 HD	Visijet M3 Crystal	6.81–34.63	(1)	[49]
BJP	X1-Lab 3D printer	Stainless Steel (SS) 420	3.65–6.61	(1)	[50]
FDM	QiDi Tech 1	Polylactic acid (PLA)	16.7 (2.6)	(2)	[51]
SLS	EOS Formiga	Polyamide (PA)	3.3 (0.3)	(2)	[51]
PJ	Objet30 Prime	VeroClear	2.3 (0.4)	(2)	[51]
SL	Structo OrthoForm	Proprietary	1.4 (0.4)	(2)	[51]
PJ	Objet260 Connex1	VeroClear	0.9–1.2	(2)	[28]
PJ	Eden250	VeroClear	0.5–2	(2)	[24]
PJ	Eden350	VeroClear	2.8–17.6	(3)	[22]
PJ	Objet260 Connex1	VeroWhitePlus	6.7–7.3	(1)	This work
PJ	Objet260 Connex1	VeroWhitePlus	0.7–2.1	(2)	This work
PJ	Objet260 Connex1	VeroWhitePlus	0.2–19.9	(3)	This work

**Table 5:** *Dev-1* surface characterization: ANOVA table for the primary response profile [µm].

Source	Sum of Squares	df	Mean Square	F-Value	*p*-Value	
**Model**	24,197.01	9	2688.56	38.85	<0.0001	*significant*
***A*—** X− ***Coordinates***	113.78	1	113.78	1.64	0.2025	
***B*—** Y− ***Coordinates***	6933.43	1	6933.43	100.18	<0.0001	*significant*
AB	26.78	1	26.78	0.39	0.5352	
$A^{2}$	17.75	1	17.75	0.26	0.6136	
$B^{2}$	135.12	1	135.12	1.95	0.1652	
$A^{2}B$	81.73	1	81.73	1.18	0.2796	
$AB^{2}$	42.89	1	42.89	0.62	0.4328	
$A^{3}$	54.76	1	54.76	0.79	0.3757	
$B^{3}$	17,050.10	1	17,050.10	246.36	<0.0001	*significant*
**Residual**	7543.82	109	69.21			
***Lack of Fit***	2171.39	15	144.76	2.53	0.0034	*significant*
***Pure Error***	5372.44	94	57.15			
**Cor Total**	31,740.84	118				
**Std. Dev.**	8.32	$R^{2}$	0.7623			
**Mean**	−0.22					

**Table 6:** *Dev-2* surface characterization: ANOVA table for the primary response profile [µm].

Source	Sum of Squares	df	Mean Square	F-Value	*p*-Value	
**Model**	11,166.12	9	1240.68	15.46	<0.0001	*significant*
***A*—** X− ***Coordinates***	328.69	1	328.69	4.10	0.0454	*significant*
***B*—** Y− ***Coordinates***	25.80	1	25.80	0.32	0.5718	
AB	9.49	1	9.49	0.12	0.7315	
$A^{2}$	265.74	1	265.74	3.31	0.0715	
$B^{2}$	3086.83	1	3086.83	38.47	<0.0001	
$A^{2}B$	1349.06	1	1349.06	16.81	<0.0001	
$AB^{2}$	4598.28	1	4598.28	57.31	<0.0001	
$A^{3}$	40.55	1	40.55	0.51	0.4786	
$B^{3}$	449.78	1	449.78	5.61	0.0196	
**Residual**	8825.91	110	80.24			
***Lack of Fit***	7635.34	14	545.38	43.98	<0.0001	*significant*
***Pure Error***	1190.57	96	12.40			
**Cor Total**	19,992.57	119				
**Std. Dev.**	8.96	$R^{2}$	0.5585			
**Mean**	1.18					

**Table 7:** *Dev-1* and *Dev-2* characterization: ANOVA table for response *T*.

Source	Sum of Squares	df	Mean Square	F-Value	*p*-Value	
**Block**	0.19	2	0.093			
**Model**	437.19	9	48.58	290.35	<0.0001	*significant*
**A Device**	87.47	1	87.47	522.79	<0.0001	*significant*
**B-Flow Rate**	275.59	4	68.90	411.80	<0.0001	*significant*
**AB**	74.13	4	18.53	110.77	<0.0001	*significant*
**Residual**	3.01	18	0.17			
**Cor Total**	440.39	29				
**Std. Dev**	0.41	$R^{2}$	0.9932			
**Mean**	2.96					

## Data Availability

The original contributions presented in this study are included in the article. Further inquiries can be directed to the corresponding authors.
